# Abnormal branching of the left lingular pulmonary artery diagnosed by three-dimensional computed tomographic angiography in a patient with lung cancer: a case report

**DOI:** 10.1186/s13019-016-0402-6

**Published:** 2016-01-20

**Authors:** Dai Sonoda, Masashi Mikubo, Kazu Shiomi, Yukitoshi Satoh

**Affiliations:** Department of Thoracic Surgery, Kitasato University School of Medicine, 1-15-1 Kitasato, Minami-ku, Sagamihara, Kanagawa 252-0374 Japan

**Keywords:** Pulmonary artery, Abnormal branching, 3D-CT, Lung cancer

## Abstract

**Background:**

In general, there are several anomalies of the pulmonary artery. The mediastinal lingular pulmonary artery is well-known for its abnormal branching from the pulmonary artery. However, other types of variation are rare.

**Case presentation:**

We herein report the case of a patient with primary lung cancer who underwent left upper lobectomy in which the lingular pulmonary artery branching from the mediastinal basal artery was demonstrated by preoperative three-dimensional computed tomographic (3D-CT) angiography.

**Conclusions:**

A preoperative evaluation using 3D-CT angiography was useful for detecting the abnormal pulmonary branching in this patient because it allowed for the precise division of the pulmonary arteries during surgery.

## Background

In general, there are several anomalies of the pulmonary artery. Video-assisted thoracic surgery (VATS) has been shown to be minimally invasive and has become a common procedure in lung surgery. Although VATS enables surgeons to obtain the details of local structures while performing the operation, the complete view of the surgical field is limited by the view of the thoracoscope [1, 2]. The decollement of the pulmonary vessels during VATS procedures is associated with a high risk of vascular injury and hemorrhage. It is of great importance to obtain precise information on the vessels as it may help to avoid such complications and ensure a safe surgery. We herein report the case of a patient who underwent resection for lung cancer in whom abnormal branching of the left lingular pulmonary artery was revealed by three-dimensional computed tomographic (3D-CT) angiography.

## Case presentation

A 73-year-old man was found to have an abnormal shadow in the apex of his left lung on a chest X-ray film in the spring of 2013. Although he underwent a bronchofiberscopic examination at a nearby hospital, a diagnosis was not reached, and he was referred to our hospital for further evaluation.

A physical examination and laboratory investigation showed no specific findings. He had a medical history of hypertension and dyslipidemia and was a smoker with a smoking index of 700.

**Fig. 1:**
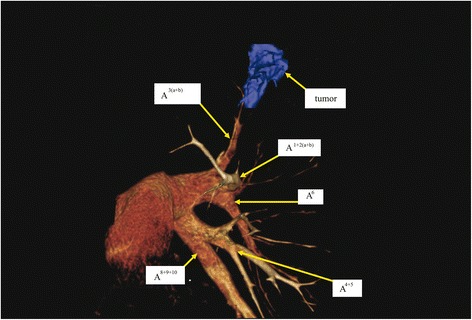
A preoperative 3D-CT angiographic image shows the left main pulmonary artery and the left mediastinal lingular and basal pulmonary arteries. The left A^4+5^ and A^8+9+10^ formed a common trunk

Chest CT revealed a solid mass measuring 32 × 15 × 16 mm in the S^1+2^ of the left lung. There was no apparent mediastinal lymph node swelling or other organ metastasis. Preoperative 3D-CT angiography with contrast medium showed that the left pulmonary A^4+5^ and A^8+9+10^ formed a common trunk (Fig. 1), branching directly from the cardiac vesicle and running anteriorly to the left lower lobe bronchus. There were no obvious abnormalities in the pulmonary veins. A bronchofiberscopic examination showed no abnormalities. On the basis of the imaging findings, left lung cancer (clinical T2aN0M0-stage IB) was suspected and he underwent surgical treatment.

**Fig. 2:**
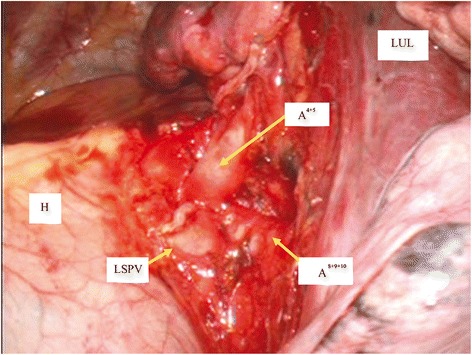
The intraoperative findings after the dissection of the upper pulmonary vein. The A^4+5^ was found to form a common trunk with the A^8+9+10^ from the front of the hilum. LUL, left upper lobe; LSPV, left superior pulmonary vein (stump), H: heart

The tumor was intraoperatively diagnosed as non-small cell lung cancer by fine-needle aspiration biopsy, and a left upper lobectomy was performed via VATS. Immediately after the pulmonary arteries were exposed from an interlobular fissure, we identified the A^6^. The A^1+2c^ and A^3a^, which branch from the A^6^ into the upper lobe, were also identified. As expected from the 3D-CT scan, the A^4+5^ formed a common trunk with the A^8+9+10^ anteriorly to the hilum (Fig. 2). The patient had an uneventful postoperative course and was discharged from hospital 10 days after surgery.

## Discussion

In the lung surgery field, there are several well-known variations in the branching pattern of the pulmonary vessels; 16.4 % of lung cancer patients have such variations [3]. The most common variation is the so-called mediastinal lingular pulmonary artery, which is the first branch of the left main pulmonary artery. Although other variations sometimes occur, it is extremely rare to encounter a lung cancer patient with a lingular pulmonary artery branching from the mediastinal basal artery. We could only find one case report in which the A^4+5+8+9b^ was reported to branch from the left main pulmonary artery [1]. In the present patient, however, the left A^4+5^ and A^8+9+10^ formed a common trunk, which was a branch from the cardiac vesicle. To our knowledge, there are no reports of this abnormality. Moreover, although bronchial abnormalities are often present in patients with vascular variations, no bronchial branching abnormalities were observed in our patient [4].

The presence of a rare pulmonary artery anomaly may result in vascular injury and bleeding during surgery, particularly when the anomaly involves the mediastinal basal segmental artery [1]. It is considered difficult to discern the mediastinal lingular and basal segmental arteries on the basis of intraoperative findings alone. Misidentification is possible, especially when an incomplete fissure or adhesion is present, because both arteries arise from the same portion and run in a similar course. In recent years, many VATS surgeons use a ‘fissureless’ technique in which the surgeon uses a stapler to first divide the hilar bronchovascular structures and then divide the main part of the fissure, in order to minimize the incidence of air leak after anatomical lung resection. With this technique, the surgeon should take care to avoid stapling the arteries and to avoid leaving a fissure, as doing so may cause severe complications such as hemorrhage or ischemia [5]. In patients with a mediastinal pulmonary artery, vascular treatment should be managed with consideration of the possibility that the mediastinal pulmonary artery may enter the lingular artery or basal segmental artery.

3D-CT has recently been shown to be effective in clearly depicting the structure of the bronchi and pulmonary vessels, allowing surgeons to understand the course of the pulmonary vessels stereoscopically. Watanabe et al. reported that the accuracy and usefulness of 3D-CT angiography may increase the safety of surgical procedures and thereby reduce surgical morbidity [2]. In that report, 3D-CT was performed in 14 patients with primary lung cancer who underwent lobectomy, and the images of the pulmonary artery branching patterns that were obtained by 3D-CT using multidetector-row CT were compared with the intraoperative findings of each case. According to that report, 98 % (84/86) of the pulmonary artery branches were successfully identified on preoperative 3D-CT. Furthermore, the two branches that were missed on 3D-CT were small vessels of less than 1.5 mm in actual diameter.

Based on the present case, 3D-CT angiography is highly recommended for the preoperative assessment of anatomic pulmonary resection because it can be performed quickly and easily and because it provides visual information on any abnormal branching of the pulmonary arteries. A careful preoperative evaluation by 3D-CT angiography enables surgeons to perform lung surgery more safely and with fewer complications.

## Conclusions

The branching patterns of the pulmonary vessels are diverse. A preoperative understanding of the course of the pulmonary vessels is important for achieving safe surgical management. 3D-CT angiography enables the surgeon to preoperatively confirm the detailed anatomy, including any abnormal branching, of the pulmonary arteries. Moreover, because of the narrow field of view provided by VATS, 3D-CT angiography provides anatomical information that may help to facilitate a safe resection.

## Consent

Written informed consent was obtained from the patient for publication of this Case report and any accompanying images. A copy of the written consent is available for review by the Editor-in-Chief of this journal.

## Abbreviations

3D-CT: three-dimensional computed tomographic
VATS: video-assisted thoracic surgery
